# Single molecule mass photometry of nucleic acids

**DOI:** 10.1093/nar/gkaa632

**Published:** 2020-08-05

**Authors:** Yiwen Li, Weston B Struwe, Philipp Kukura

**Affiliations:** Physical and Theoretical Chemistry Laboratory, Department of Chemistry, University of Oxford, South Parks Road, Oxford OX1 3QZ, UK; Physical and Theoretical Chemistry Laboratory, Department of Chemistry, University of Oxford, South Parks Road, Oxford OX1 3QZ, UK; Physical and Theoretical Chemistry Laboratory, Department of Chemistry, University of Oxford, South Parks Road, Oxford OX1 3QZ, UK

## Abstract

Mass photometry is a recently developed methodology capable of measuring the mass of individual proteins under solution conditions. Here, we show that this approach is equally applicable to nucleic acids, enabling their facile, rapid and accurate detection and quantification using sub-picomoles of sample. The ability to count individual molecules directly measures relative concentrations in complex mixtures without need for separation. Using a dsDNA ladder, we find a linear relationship between the number of bases per molecule and the associated imaging contrast for up to 1200 bp, enabling us to quantify dsDNA length with up to 2 bp accuracy. These results introduce mass photometry as an accurate, rapid and label-free single molecule method complementary to existing DNA characterization techniques.

## INTRODUCTION

Single molecule analysis has had a tremendous impact on our ability to study DNA structure, function and interactions (1). Next generation sequencing heavily relies on single-molecule methods, be it using single molecule fluorescence (2,3) or nanopore-based approaches (4,5). Similarly, single molecule methods are now extensively used in a variety of incarnations to study DNA–protein interactions (6), with both DNA and proteins visualized by fluorescence labelling to reach single molecule sensitivity (7). Label-free detection and quantification would be highly desirable in this context due to the associated reduction in experimental complexity and minimization of potential perturbations. While visualization of single DNA molecules has been possible for decades using non-optical methods, such as electron microscopy (8) and atomic force microscopy (9), which can also be used to study mechanical properties (10), label-free optical detection has remained a considerable challenge.

Label-free detection of single proteins has been reported for the first time in 2014 (11,12) in the context of increasing sensitivity of interferometric scattering microscopy (13,14). Further improvements to the detection methodology (15), recently lead to the development of mass photometry (MP), originally introduced as interferometric scattering mass spectrometry (16), which enables not only label-free detection and imaging of single molecules, but critically their quantification through mass measurement with high levels of accuracy, precision and resolution at a lower detection limit on the order of 40 kDa. Given that biomolecules have broadly comparable optical properties in the visible range of the electromagnetic spectrum (17,18), we therefore set out to investigate to which degree the capabilities of MP translate to nucleic acids, which would enable not only their detection, imaging and analysis, but also provide a universal route to studying protein-DNA interactions at the single molecule level.

**Figure 1.**
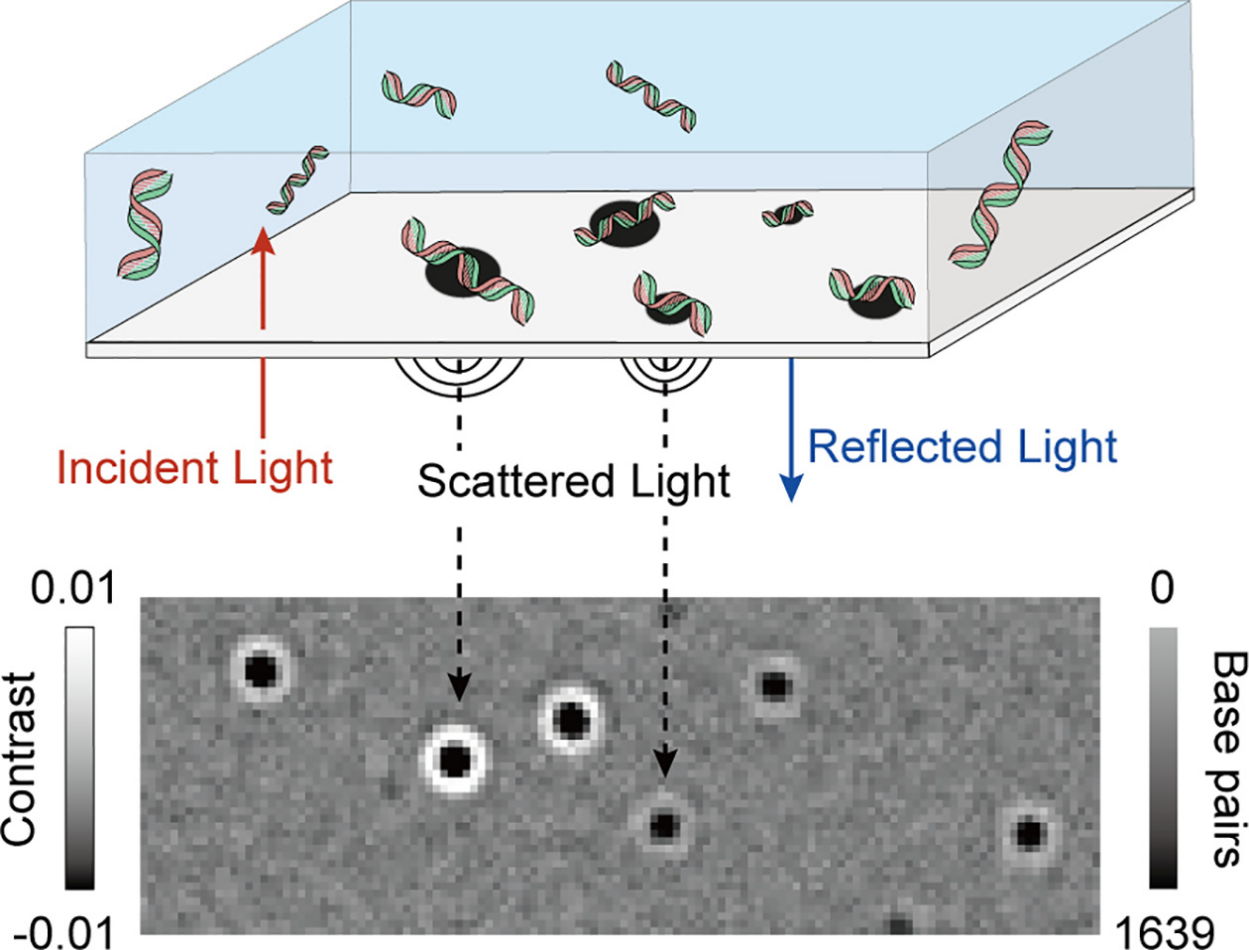
Working principle of label-free DNA detection and quantification by mass photometry. Individual DNA molecules diffusing in solution bind to an appropriately charged glass surface. Binding events cause changes to the reflectivity of the interface, visualized by a contrast-enhanced interferometric scattering microscope through the interference between scattered and reflected light.

The operating principle behind MP is based on accurately measuring the change in reflectivity of a glass-water interface caused by interference between light scattered by a molecule binding to the interface and light reflected at that interface (Figure 1). The experiment involves placing a small droplet of sample solution on top of microscope coverglass, to which molecules bind non-specifically, although in the case of DNA appropriate charging of the glass surface is required to achieve tight binding (see Materials and Methods). We then visualize individual binding events on top of the static imaging background caused by residual substrate roughness by computing the differences between batches of averaged reflectivity images, which leads to the appearance and disappearance of single molecule signals from irreversible binding events in a continuous recording (Supplementary Movie S1) (12,15). By determining the point in time when each individual molecule binds, we can then quantify the associated reflectivity change, yielding highly accurate, precise and resolved contrast distributions.

## MATERIALS AND METHODS

Solvents and chemicals were purchased from Sigma Aldrich unless otherwise noted. Milli-Q water and high-grade solvents were used for all experiments. A double-stranded DNA ladder consisting of 100, 200, 400, 800, 1200 and 2000 base pairs was purchased from Invitrogen (Cat. No. 10068013). A 100 bp dsDNA ladder consisting of 13 individual chromatography-purified DNA fragments in the range of 100–2000 bp was also purchased from Invitrogen (Cat. No. 15628050). Circular single-stranded DNA samples with 4536, 6048, 7249, 8064 bases were prepared as previously described (19). Samples were kept in TE buffer and no denaturing agent was used during the measurements. Single stranded DNA (155-mer) was synthesized on an Applied Biosystems 394 automated DNA/RNA synthesizer using a standard 0.2 μmole phosphoramidite cycle of acid-catalysed detritylation, coupling, capping and iodine oxidation. Stepwise coupling efficiencies and overall yields were determined by the automated trityl cation conductivity monitoring facility and was >98.0%. Standard DNA phosphoramidites and additional reagents were purchased from Link Technologies Ltd, Sigma-Aldrich, Glen research and Applied Biosystems Ltd. All beta-cyanoethyl phosphoramidite monomers were dissolved in anhydrous acetonitrile to a concentration of 0.1 M immediately prior to use with a coupling time of 50 s. Cleavage and deprotection were achieved by exposure to concentrated aqueous ammonia solution for 60 min at room temperature followed by heating in a sealed tube for 5 h at 55°C. Purification was carried out by denaturing 8% polyacrylamide gel electrophoresis, without any dye. In brief, formamide (500 μl) was added to the DNA sample (500 μl in water) before loading to the gel, bands corresponding to the full length were excised and the DNA was isolated using the ‘crush and soak method’. The excised polyacrylamide pieces were broken down into small pieces then suspended in distilled water (25 ml). The suspension was shaken at 37°C for 18 h, then filtered through a plug of cotton wool. The filtrate was concentrated to ∼2 ml, then desalted using two NAP-25 columns followed by one NAP-10 column. The desalted eluent was lyophilized prior to use.

Prior to MP measurements, 231 nM (0.1175 μg/μl) double-stranded DNA stock solutions were diluted 25-fold in 5 mM Tris, 10 mM MgCl_2_, pH 8. The 100 bp dsDNA ladder stock solutions (0.5 μg/μl) were diluted 200-fold. Single-stranded DNA stock solutions (167 nM for 4536 nt, 125 nM for 6038 nt, 100 nM for both 7249 nt and 8064 nt) were diluted 10-fold in the same buffer. 7.79 μM 155 nt single-stranded DNA stock solutions were diluted 1000-fold. Standard protein marker solutions were diluted 10-fold in the same buffer. Samples were kept at room temperature during analysis.

### Mass photometry

Microscope coverglass (24 × 50 mm # 1, 5 SPEZIAL, Menzel-Glaser) and (3-aminopropyl)triethoxysilane (APTES)-functionalized coverslips were prepared as described previously (16,20). Briefly, coverslips were cleaned by sequential sonication in 2% Hellmanex (Hellma Analytics), water and iso-propanol for 10 min before plasma cleaning with oxygen (Diener electronic Zepto) for 8 min. The coverslips were then immersed in 200 ml 2% APTES solution in acetone for 1 min with agitation before rinsing in 200 ml acetone. Finally, the coverslips were incubated at 110°C for 1 h and cleaned by sonication in isopropanol (10 min) and water (5 min) before drying under a nitrogen stream.

Mass photometry was performed using a home-built microscope as previously described (15,16) and illustrated in Supplementary Figure S1. Instrument settings were as follows: Laser wavelength: 520 nm, laser power: 300 mW, frame rate = 955 Hz, exposure time = 998 μs, temporal averaging: 5-fold, pixel binning: 4 × 4, field of view: 3.5 × 10 μm. This leads to an effective frame rate of 191 Hz and an effective pixel size of 84.4 nm. All measurements were performed using flow chambers made by microscope cover glass and double-sided tape with 15 μl sample per analysis. Flow chambers were first filled with a buffer blank to position the coverslip into the optimal focus position. Samples were then added to one side of the flow chamber and introduced by capillary flow with the aid of tissue paper to draw liquid into the chamber. Data acquisition was started within 15 s of sample addition for a total of 120 s. In total, five replicates were taken for the double-stranded DNA ladder and three replicates for each single-stranded DNA sample. Data acquisition was performed using custom software written in LabView, generating a single movie file (.tdms) for further analysis. Three replicates were taken for the standard protein marker and five replicates were taken for the 100 bp double-stranded DNA ladder.

### Data analysis

All acquired movies were processed and analysed using Discover MP v1.2.4 (Refeyn Ltd). The analysis procedure involved two fitting parameters for identifying landing events: (i) Threshold 1 related to a given particle contrast amplitude relative to the background and (ii) Threshold 2 related to the radial symmetry of the detected point spread function (PSF) of the same particle.

Analysis parameters for dsDNA and ssDNA samples are shown in Table 1. Threshold 1 was set to 1 to quantify smaller DNA (100 bp dsDNA and 155 nt ssDNA). In the presence of background due to buffer impurities or other contaminants, Threshold 1 was increased to 3 where necessary, which was informed by running buffer blanks. For larger DNA samples, Threshold 1 = 1 provided effectively indistinguishable results to Threshold = 3, since changes in this value only affected the low size regime (<300 kDa). With Threshold 2, values were set to the default (0.2) and slightly greater values (0.3) were chosen for longer DNA strands to allow for any deformations of the PSF due to species exceeding the diffraction limit, resulting in a slightly asymmetric point spread function. In the case of the linear dsDNA ladder, using the same Threshold 2 for analysing 100 and 2000 bp was not ideal, especially considering the size of 2000 bp dsDNA was approaching the diffraction limit. The shape of the point spread function of a 2000 bp DNA became asymmetric. However, we found no evidence that the value of Threshold 2 has a measurable effect on the contrast of the landing event, either in DNA or protein measurements.

**Table 1.** Analysis parameters for dsDNA and ssDNA samples

DNA sample	Number of binned frames, ***n***	Threshold 1	Threshold 2
dsDNA ladder	8	1	0.2
ssDNA 155 nt	8	1	0.2
ssDNA 4536 nt	5	3	0.3
ssDNA 6048 nt	5	3	0.3
ssDNA 7249 nt	5	3	0.3
ssDNA 8064 nt	5	3	0.3

The output files contained a list of all detected particles within the analysed movie and their corresponding contrast values. The contrasts of all landing events were plotted as a scatter plot along the time axis. A histogram of the number of landing events and the contrasts was then generated. The resulting peaks were fit to a sum of Gaussians and the mean of the fitted peaks was taken as the contrast for each DNA component. The contrast to base pair ratio was determined by a linear fit. Base pair error was given as a deviation of the measured number of base pairs from the nominal number given by the manufacturer. The contrast to nucleotide ratio and the nucleotide error for ssDNA was determined in a similar manner. The input values used to calculate the estimator bandwidth (bw_method) in the violin plots using the python package matplotlib (both the base pair error and nucleotide error) were set to 0.32.

### Diffusion correction and concentration measurement

The relative abundance of each DNA fragment in the dsDNA ladder was calculated from the area of each Gaussian peak in the kernel density estimate (KDE) plot, ${\rm{a}} = {\rm{A}}\sigma \sqrt {2{\rm{\pi }}}$, where *a* is the area, *A* is the maximum height at centroid and *σ* is the standard deviation of the fitted Gaussian. The contrast magnitude achievable with the 100 bp species approached the limit where the instrumental readout in terms of counting molecules is quantitative.

To account for differences in binding rates and thus molecule counts caused by varying diffusion speeds, we applied a correction to the measured mass distributions (16). We assumed that the binding rate constant scales with the diffusion coefficient, which has been reported to be roughly proportional to (base pair)^−0.72^ for DNA (i.e., ${k_i} = {\rm{\alpha *}}b{p^{ - 0.72}}$, where *k_i_* is the binding rate constant for DNA component *i* and *α* is a scaling factor) (21). We assumed that the scaling factor *α* is constant for all DNA components. To estimate the scaling factor *α*, an exponential function was fitted to the number of landing events *vs* time resulting in an average binding rate, *k*. The scaling factor was calculated as: $\alpha = \frac{{\rm{k}}}{{ < {\rm{bp}}{ >^{( - 0.72)}}}}$, where <bp> is the average number of base pairs of all the DNA components in solution calculated based on the distribution of each DNA component, $ < {\rm{bp}} >= \frac{{\sum\nolimits_{{\rm{i\ }} = {\rm{\ }}1}^{\rm{N}} {{\rm{b}}{{\rm{p}}_{\rm{i}}}{\rm{*}}{{\rm{a}}_{\rm{i}}}} }}{{\rm{N}}}$, where *bp_i_* is the number of base pairs and *a_i_* the relative abundance measured experimentally of DNA component *i*, and *N* is the total number of species in solution. To accurately estimate the proportion of each DNA fragment in solution, it is important to account for landing events that occur between the time when the sample is added (*t*_addition_) and when data acquisition starts (*t*_0_). We accounted for this by fitting the exponential decay of measured binding events (from *t*_addition_) from the addition of sample to completion of all sample binding (*t* = infinity) (Supplementary Figure S2). Experimentally, we integrated from a given time *t*_0_ = 15 s after addition of sample up to a later time, *t*_final_ = 135 s, when the acquired movie ended. Relating these two, the corrected intensity was given by: ${{\rm{a^{\prime}}}_{\rm{i}}}{\rm{ = }}{{\rm{a}}_{\rm{i}}}\frac{{{{\rm{e}}^{({{\rm{k}}_{\rm{i}}}{{\rm{t}}_0})}}}}{{1 - {{\rm{e}}^{ - {{\rm{k}}_{\rm{i}}}({{\rm{t}}_{{\rm{final}}}} - {{\rm{t}}_0})}}}}$, where *a’_i_* is the intensity of DNA component *i* corrected over all time, and *a_i_* is the experimentally measured abundance. The corrected mass distribution was then renormalized.

## RESULTS

**Figure 2.**
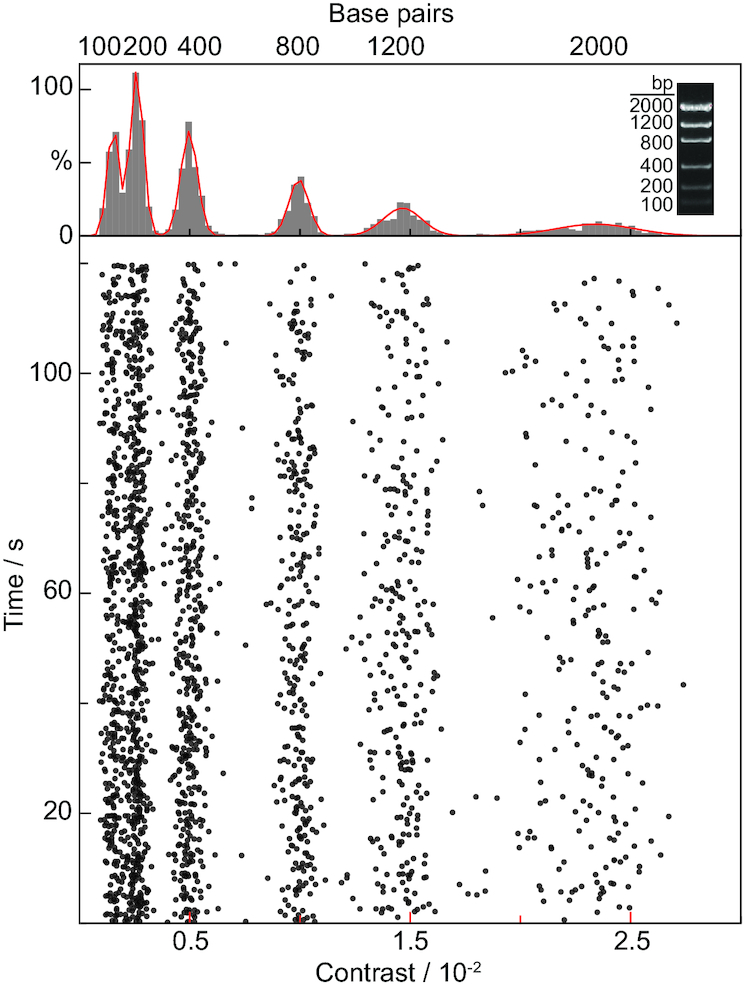
Scatter plot and resulting contrast histogram (with corresponding kernel density estimate shown in red) obtained by quantifying the image contrast on a molecule-by-molecule basis for a low mass dsDNA ladder (see inset). The close correspondence between the gel and resolvable features in the contrast histogram allows for assignment by inspection.

To test the applicability of MP to a representative DNA sample, we started with an analysis of a standard low mass dsDNA ladder. A 9 nM solution led to distinct molecular binding events with clearly varying molecule-to-molecule contrasts (Supplementary Movie S1), while frequent unbinding events were observed on non-APTES coverslips (Supplementary Movie S2), suggesting that appropriate surface charge was required to achieve tight surface binding. The contrast histogram of the landing events on non-APTES coverslips showed very poor resolution (Supplementary Figure S3). As for signals collected on APTES coverslips, a scatter plot of these signals obtained by quantifying the signal magnitude for each individual binding event exhibited six clear bands, as expected from the ladder used (Figure 2). This separation persisted upon binning into a contrast histogram, with baseline resolution from the second peak onwards. The observed spacing and knowledge of the ladder composition allowed for assignment to different contour lengths by inspection. These results demonstrate that MP can detect single DNA molecules without labels with a comparable performance in terms of mass sensitivity and resolution to polypeptides.

**Figure 3.**
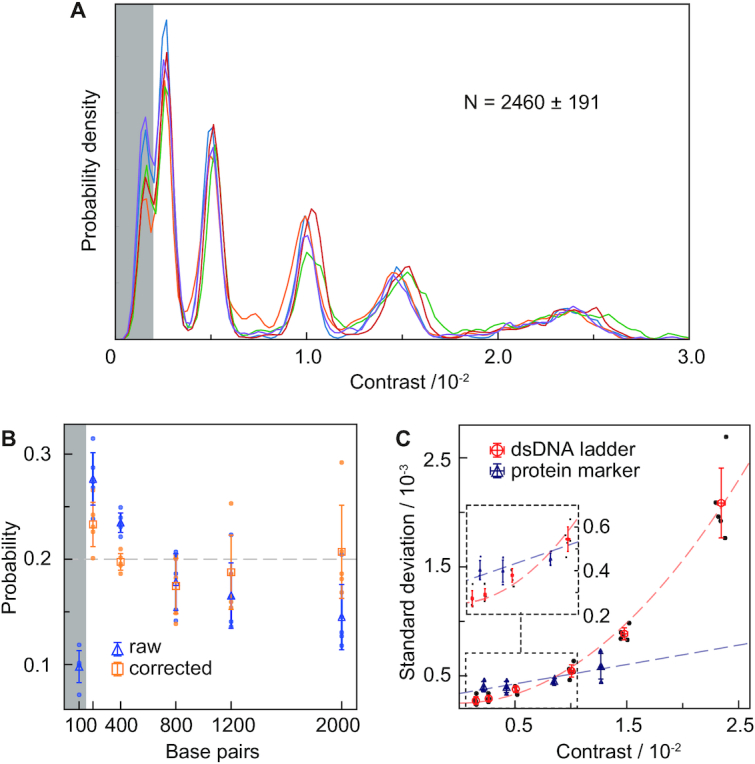
Achievable concentration precision and base pair resolution. (**A**) Reproducibility of individual MP measurements of the same dsDNA ladder sample. The plots were generated from mass histograms using a Kernel Density of width 2.1 × 10^–4^. (**B**) Extracted mole fractions before and after correction for length-dependent diffusion. (**C**) Comparison of contrast resolution between dsDNA and a globular protein mixture of comparable imaging contrast for the same instrument.

Quantifying the landing frequency should provide direct information on molecular concentration for each species assuming label-free, universal detection of all binding events. Multiple repeats of the ladder experiment exhibited high reproducibility (7.7% RMS) in the total number of detected molecules, despite the simplicity and inherent variability of the measurement due to manual sample addition and timing when recording was started (Figure 3A). The relative fluctuations between the peak areas amounted to 12 ± 3.3% RMS (Figure 3B, blue dots). Despite the fact that the ladder contains an equimolar mixture of molecules, we observed clear variations in peak areas with a drop towards larger species. The landing frequency of molecules with the surface, and thus detection rate, however, is not only a function of solution concentration, but also diffusion coefficient, which decreases considerably with contour length (21). Our qualitative observation of a decrease in binding events with contour length agreed with this expectation.

To account for these differences, we needed to relate the number of molecules that have bound during our finite measurement window to the number we would have observed for an infinite observation time where all molecules would be depleted from solution by surface binding. Since smaller molecules diffuse more quickly, more of them will be removed from solution initially, resulting in a concentration difference once the measurement was started, after sample application, compared to the original solution (16). We therefore applied a correction factor to account for sampling occurring before and after the recorded measurement (Supplemental Figure S2). The duration of the measurement itself also influenced how the correction is applied, such as with longer movies, which will change the balance towards longer DNA. (Supplemental Figure S4). Correcting for this behaviour increased the amount of large relative to small molecules (Figure 3B, orange dots), in this case resulting in effectively equimolar concentrations for all species of contour length 200 bp or larger. The lower than expected concentration of 100 bp species was most likely caused by non-unity detection efficiency as discussed previously.

Careful inspection of the obtained mass distribution in Figure 3A revealed a clear variation in peak width with molecular size, with lower widths for small species and much larger widths for large species compared to globular proteins producing similar imaging contrast (Figure 3C). Given that we found no evidence of binding mode or other measurement differences that could cause variations in contrast, the only explanation for the observed differences in peak widths for similar optical contrast must be due to molecules occupying different areas on the surface in a way that becomes significant (>50 nm) for our interferometric measurement (14) in the light of the diffraction limit (200 nm), and the comparatively high spatial confinement of oligomeric proteins (≪50 nm). We would expect this effect to become relatively more pronounced for longer DNA molecules in the light of the persistence length of DNA (∼150 bp), in line with our observations. Similarly, the reduced width for small species (<400 bp) can be explained by a comparatively lower degree of disorder in terms of structure and thus polarizability given the structural rigidity of DNA on short length scales compared to globular proteins binding non-specifically to a glass surface. Such variability in surface adhesion agrees with observations of DNA conformations on mica surfaces observed by AFM (22,23).

**Figure 4.**
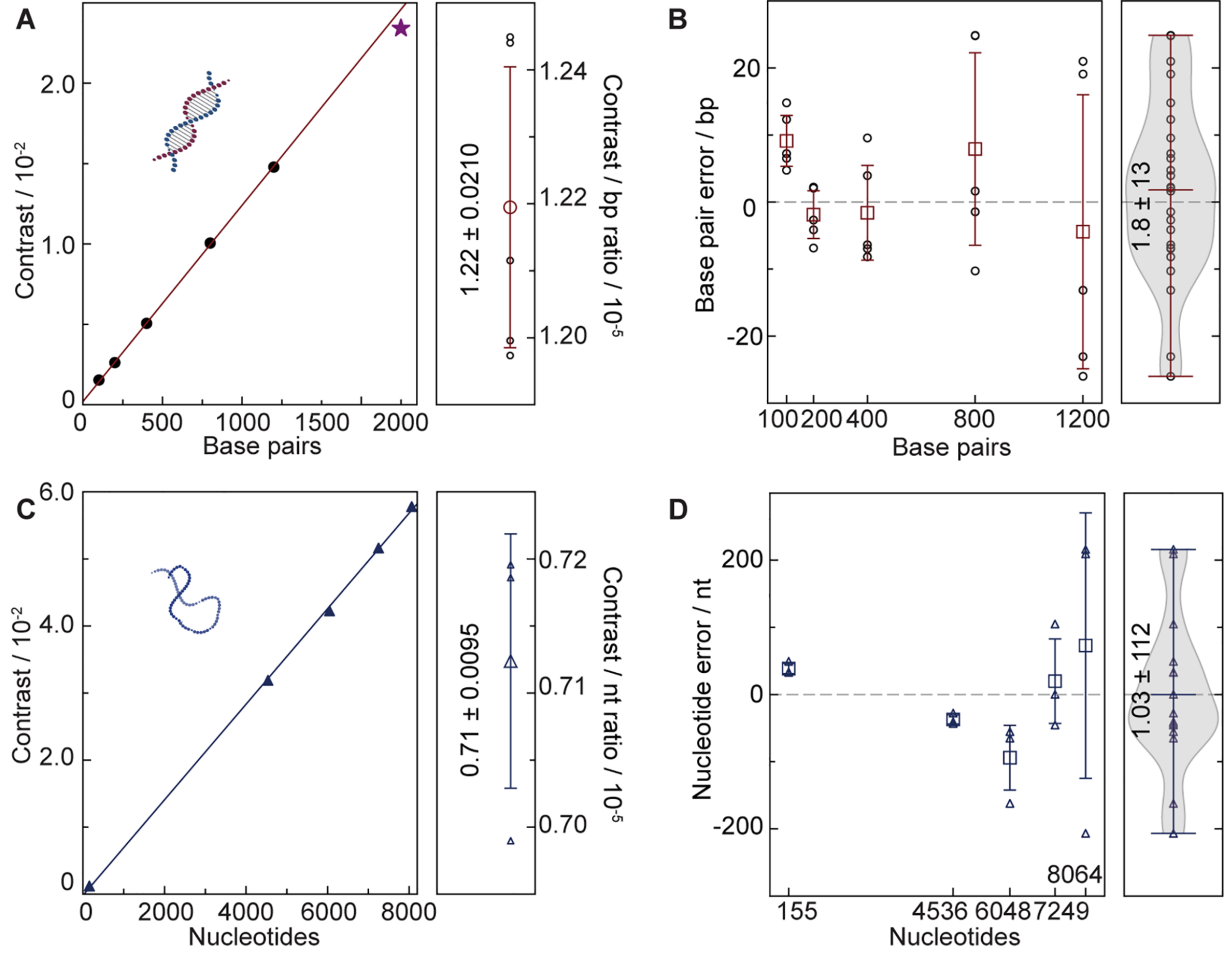
Characterization of nucleotide accuracy and precision for dsDNA and ssDNA. (**A**) Correlation between imaging contrast and number of basepairs. The 2000 bp data point (star) was omitted for the calibration due to the molecular size becoming comparable to the diffraction limit (200 nm). (**B**) Resulting base pair accuracy for independent measurements using the average contrast-to-bp conversion. (**C**, **D**) Equivalent measurements for ssDNA.

The observed peak spacing roughly matched the spacing expected for a direct proportionality between the MP contrast and the number of base pairs. To quantify this correlation, we repeated these measurements 5 times, finding almost perfect correspondence (*R*^2^ = 0.9998 ± 0.0001) for all species up to 1200 bp, with a slightly lower than expected contrast for the largest (2000 bp) species (Figure 4A, left). Given that the repeats were performed on different substrates, these results demonstrate the high repeatability of the measurement, in line with our original results on globular proteins (16). The resulting conversion from imaging contrast to contour length amounts to 1.22 ± 0.02 × 10^–5^/bp for dsDNA. Applying this conversion factor to each individual measurement allowed us to determine the average base pair error, which amounted to 1.8 ± 13 bp up to 1200 bp, with slight variations as a function of molecular size (Figure 4B). Characterizing another commercial dsDNA ladder showed a contrast-to-bp ratio of 1.20 ± 0.0208 × 10^–5^/bp, which was within the experimental error of our initial measurements (Supplementary Figure S5). At this stage, it is unclear to which degree the observed error is indeed representative of the limits achievable by MP or whether they are caused by sequence-specific variations in molecular mass or molecular polarizability, which we could not account for given that the sequence of the ladder components was unknown.

Repeating the same process with ssDNA revealed a similar linear relationship between the number of bases and the imaging contrast, without noticeable deviations for larger species (Figure 4C). The associated average nucleotide error of 1 ± 112 nt was much larger (Figure 4D), although this is may have been partially caused by the requirement to run separate experiments for each species, rather than mixtures as for dsDNA due to insufficient sample purity. In order to compare the contrast-to-bp ratio between ssDNA and dsDNA, the contrast-to-nt ratio of ssDNA had to be multiplied by 2, resulting in 1.42 ± 0.0190 × 10^–5^/bp, which was slightly larger than dsDNA. This was likely because of a higher effective density of ssDNA in the presence of Mg^2+^ arising from increased flexibility or polarizability in the absence of basepair hybridization (23). The lack of non-linear contrast behaviour even for very long ssDNA molecules was likely due to the fact that ssDNA becomes highly compacted under the buffer conditions used, ensuring essentially uniform densities for all studied species irrespective of the number of bases in contrast to dsDNA, where the contour length of DNA played a non-negligible role.

## DISCUSSION

Taken together, these results establish mass photometry as a novel analytical approach for studying nucleic acids, with a range of potential future analytical applications, albeit with some intrinsic limitations. Base pair resolution will likely never reach that achievable with electrophoretic methods, but the solution operation of MP provides much potential for combination with such approaches in the future to improve the resolution beyond what can be achieved by manual sample addition. The ultimately achievable basepair accuracy is generally subject to the underlying mechanism responsible for the optical contrast we measure, which is the molecular polarizability. This, in turn, depends on the optical properties of the scatterer, as well as that of its environment, which can become complex especially on the nanoscale. As a result, it is in principle possible that errors would arise due to factors such as the level of GC content, whether DNA is nicked, supercoiled, or circular, as well as effects due to variations in secondary structure. All of these factors may indeed limit the performance of MP for how accurately unknown samples can be characterized, which will require a more in-depth study encompassing a much broader set of samples.

Notwithstanding these limitations, there remain a number of unique advantages of MP for studying nucleic acids and their interactions. We have demonstrated both absolute and relative concentration measurements based on molecular counting with comparable precision to UV-absorption based approaches, but with the specific advantage of operation at low concentrations (nM) and minimal sample requirements, currently only limited by our sample delivery approach. The observed base pair accuracy of 2 bp is comparable to unreferenced capillary electrophoresis, and could be in principle improved further by using appropriate internal standards. Furthermore, mass photometry has proven surprisingly robust to even quite significant variations in molecular identity, while showing fairly little sensitivity to structure, such as quantifying changes in the number of lipids in lipid nanodiscs (16). Our observation of comparable mass-to-contrast ratios between proteins (2.35 ± 0.01 × 10^-5^ /kDa) and DNA (ss: 2.19 ± 0.03 × 10^-5^ /kDa, ds: 1.88 ± 0.03 × 10^-5^ /kDa) confirms this rough assessment, meaning that DNA and polypeptides can be simultaneously quantified with a mass accuracy sufficient for many applications aimed at protein-DNA interactions, in particular when separate calibrations are available. It also and has so far been largely insensitive to molecular structure, e.g. when comparing molecules of very different shapes such as largely spherical proteins with antibodies (24). While it is difficult at this stage to predict the exact levels of sensitivity of MP contrast to structural details, the linearity of contrast with the number of nucleotides presented herein is in line with the accuracy limits of MP (∼2% of object mass) irrespective of molecular shape or conditions.

Given the single molecule nature of MP, coupled with its intrinsic compatibility for visualizing and quantifying proteins, and suitability for combination with single molecule fluorescence imaging (25), MP is likely to become a powerful addition to the existing toolbox of single molecule methodologies aimed at quantifying and studying nucleic acids and their interactions.

## Supplementary Material

gkaa632_Supplemental_Files
