# Supplementary material for: Single molecule mass photometry of nucleic acids
Source: Nucleic Acids Res. 2020 Aug 5;48(17):e97. doi: 10.1093/nar/gkaa632 (PMC7515692; doi:10.1093/nar/gkaa632)
Supplement: gkaa632_Supplemental_Files [file gkaa632_supplemental_files.zip › Supplementary Information.docx]

**Supplementary Information**

**Single molecule mass photometry of nucleic acids**

Yiwen Li^1^, Weston B. Struwe^1^ and Philipp Kukura^1,^*

^1^ Physical and Theoretical Chemistry Laboratory, Department of Chemistry, University of Oxford, South Parks Road, Oxford OX1 3QZ, United Kingdom

**Supplementary Figures**


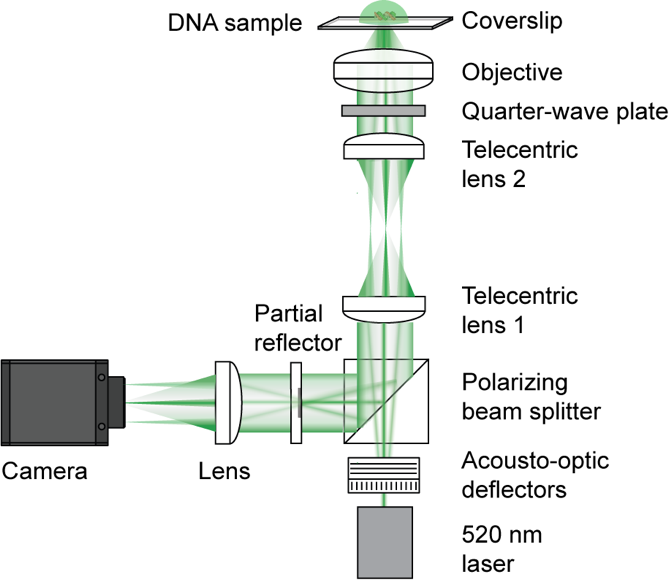


**Supplementary Figure S1: The schematic of the instrumental setup.** The home-built experimental mass photometry setup is identical to that described in (15), except that the operating wavelength is 520 nm. The setup based on acousto-optic scanning of a collimated beam using a telecentric imaging setup. The combination of a λ/4 waveplate and polarizing beam splitter separates incident from scattered and reflected light. The two lenses also create an image of the back focal plane of the microscope objective where the partial reflector attenuates the back-reflected beam while leaving scattered light effectively unchanged. A final lens forms an image on the camera. The optics are mounted to a thick aluminum plate and fully enclosed to minimise the effects of vibrations and airflow on the measurement.


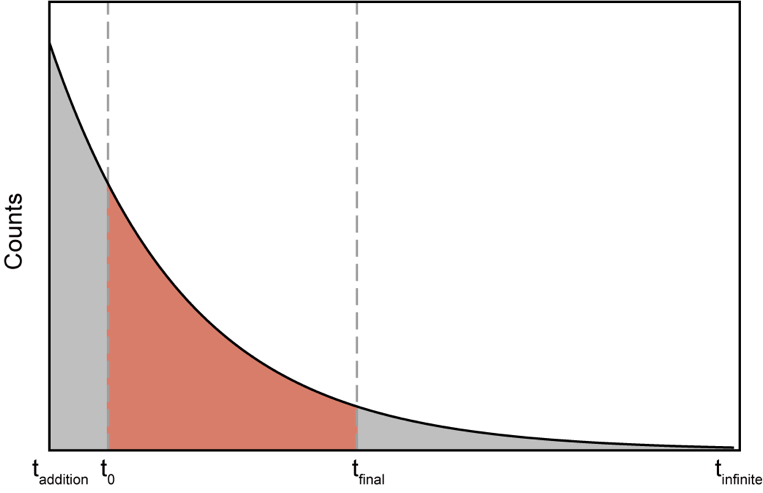


**Supplementary Figure S2: Illustration of diffusion correction.** The diffusion correction is based on determining the total number of particles that land on the glass coverslip (i.e. detector) before and after the recorded measurement. Knowledge of the number of particles measured during the finite measurement time window (from *t_0_* to *t_final_*, red area) enables us to determine the total number of particles in solution (gray area) by applying an integration from *t_addtion_* (when the sample is applied to the instrument) to *t* = ∞ . The correction factor is given by $\frac{e^{k_{i}t_{0}}}{1-e^{-k_{i}(t_{\mathrm{final}}-t_{0})}}$, where *k_i_* is the binding rate constant for DNA component *i*.


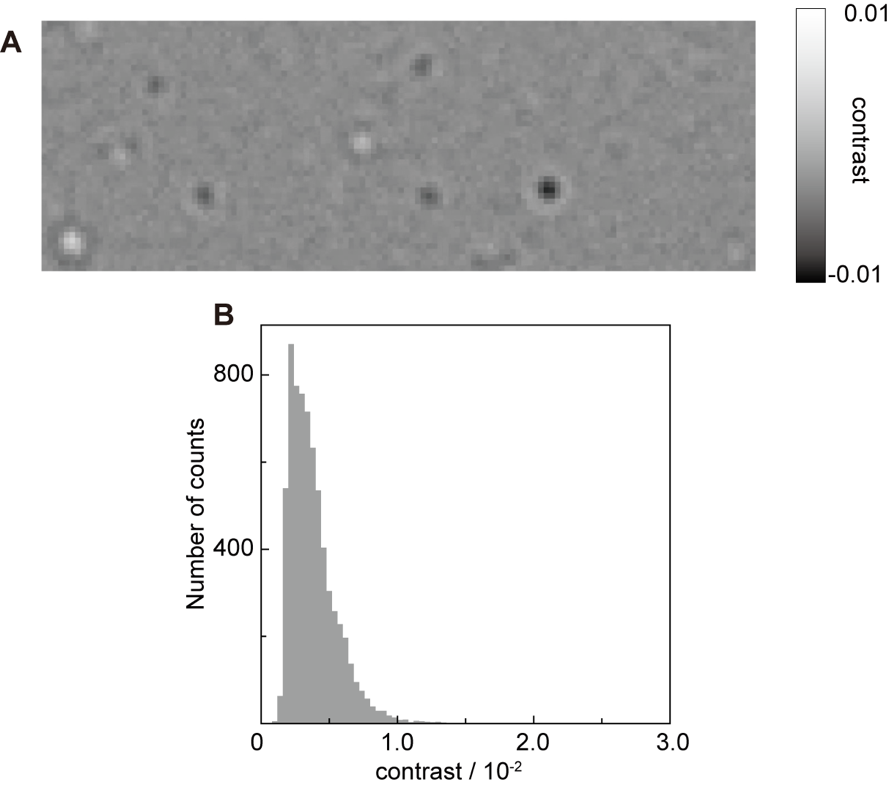


**Supplementary Figure S3:** Histogram of dsDNA ladder binding to a regular glass coverslip.


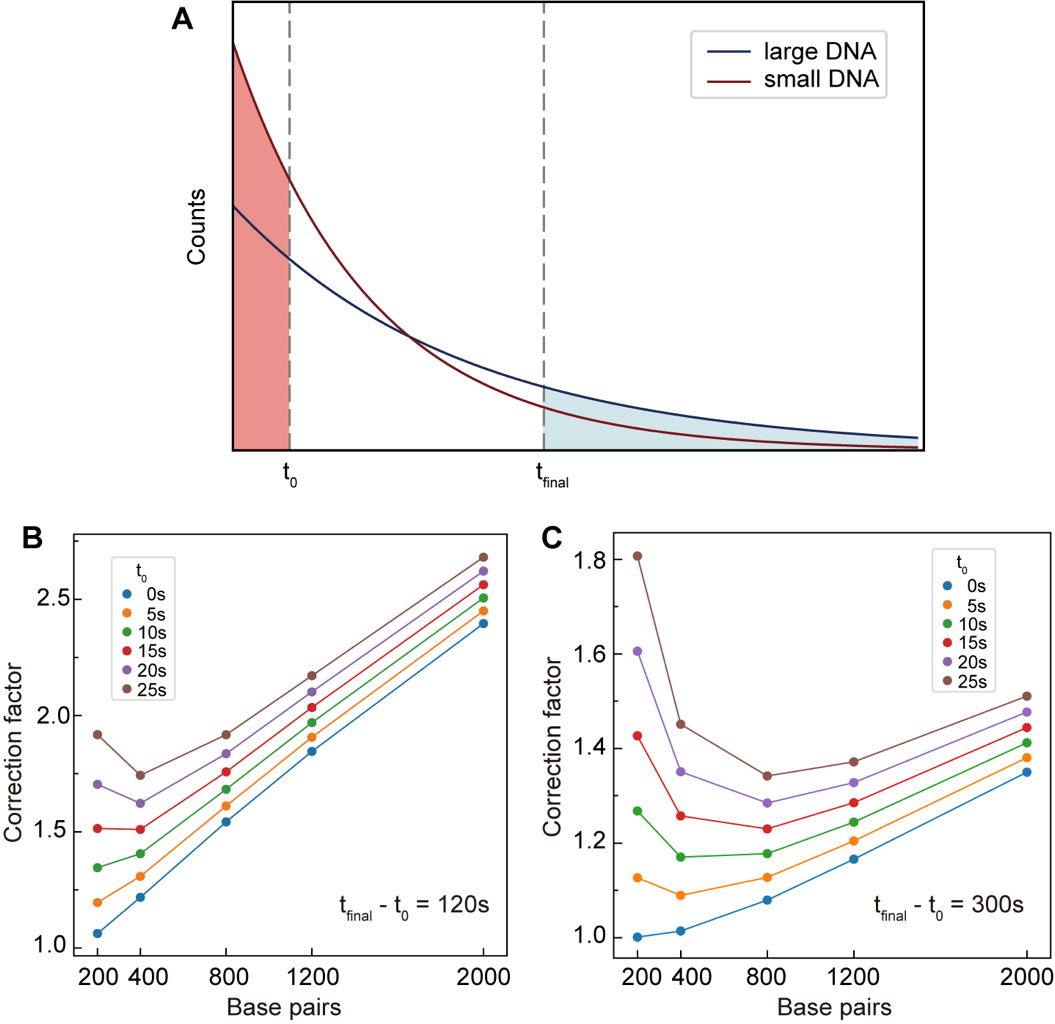


**Supplementary Figure S4: Comparison of diffusion correction as a function of strand length. A,** Exponential decay of the binding frequency for both large DNA strands and small DNA strands starting nominally at the same concentration. The later the mass photometry recording begins (*t_0_* is larger), more small particles are lost before the measurement starts (i.e. the red area becomes larger), resulting in larger correction factors for short vs long DNA. By contrast, in longer movies (*t_final_ - t_0_* is larger), one would collect more long DNA strands relative to the short ones (blue area gets smaller), resulting in a relatively smaller correction factor for large DNA. **B,** Correction factors when *t_final_ - t_0_* is 120s. *t_0_* ranges from 0s to 25s. **C,** Correction factors when is *t_final_ - t_0_* is 300s. *t_0_* ranges from 0s to 25s.


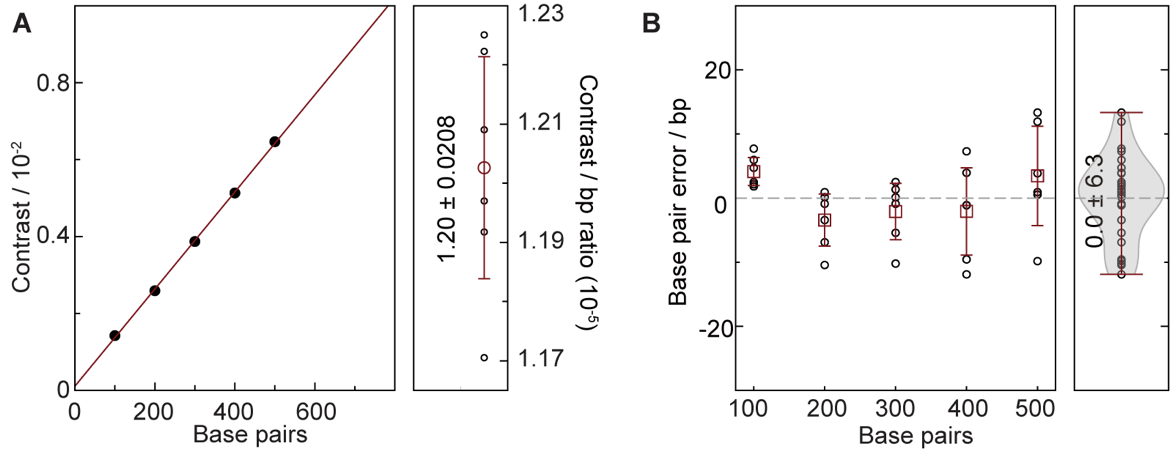


**Supplementary Figure S5: Characterisation of nucleotide accuracy and precision for a second 100bp dsDNA ladder.** **A,** Correlation between imaging contrast and base pair number. Peaks were observed at 100 bp, 200 bp, 300 bp, 400 bp and 500 bp. Larger species in the ladder were not detected because their concentrations in the ladder were too low in comparison. **B,** Resulting base pair accuracy using the average contrast-to-bp conversion.

**Supplementary Movies**


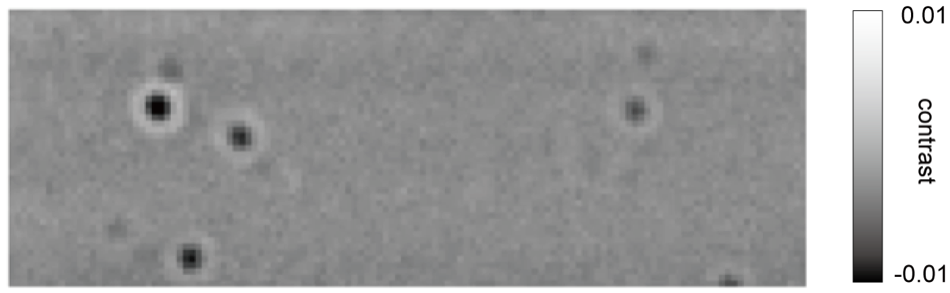


**Supplementary Movie S1:** Ratiometric movie of the dsDNA ladder binding to an APTES functionalised coverslip.


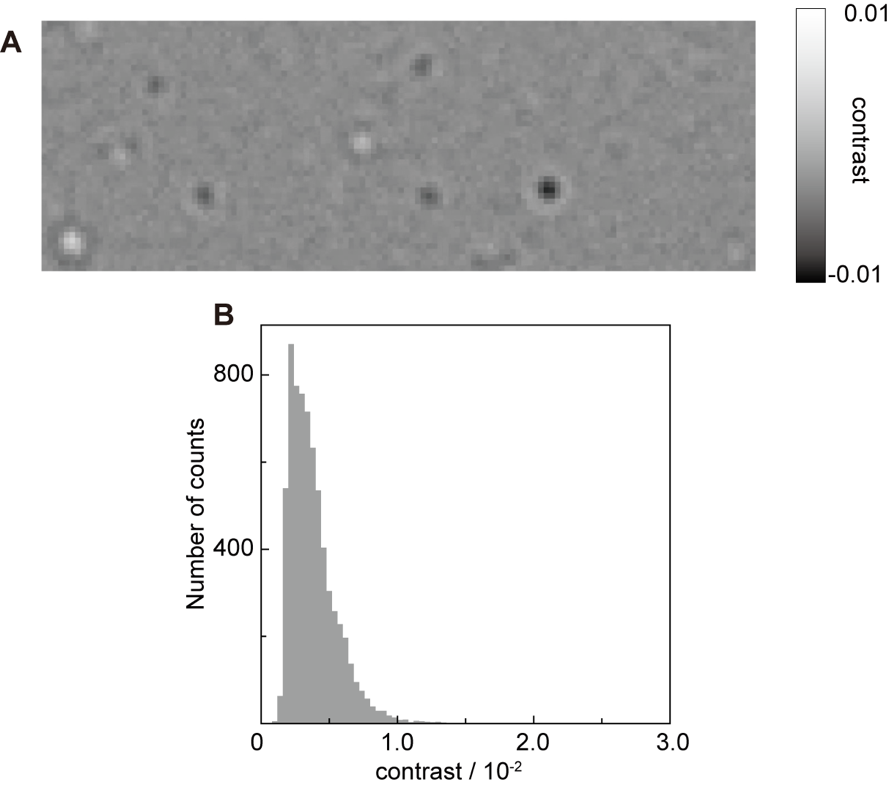


**Supplementary Movie S2: A.** Ratiometric movie of dsDNA ladder binding to a regular glass coverslip.
